# Medical Etiquette; or an Essay upon the Laws and Regulations Which Ought to Govern the Conduct of Members of the Medical Profession in Their Relation to Each Other, &c.

**Published:** 1840-07

**Authors:** 


### ART. XI.

*Medical Etiquette; or an Essay upon the Laws and Regulations which ought to govern the conduct of Members of the Medical Profession in their relation to each other, &c.* By ABRAHAM BANKS, Esq., M.R.C.S.L. &c.—*London*, 1839. 12mo, pp. 104.

THE title of this work is not strictly appropriate. The term *etiquette* relates only to manner and external observances, whereas some of the points here discussed involve the principles of professional morality, and come under the head of what is usually denominated medical ethics, while others have reference to the organization of the profession, the rights of medical practitioners, and other matters constituting what has of late years been called medical politics. We see no objection, however, to viewing these subjects in a common light. The state of manners has an intimate relation to that of morals, though not always of a direct or obvious kind, since it must be admitted that what is called good breeding often springs from the most corrupt moral sources, while the ingenuous expression of the best feelings of our nature would occasionally give rise to outrageous infractions of politeness. Still we think it may be assumed as a general position, that good manners, in the truest sense of the term, are indications of benevolent feeling and of an intelligent and cultivated mind; and this may be more especially maintained with regard to the manners of a profession which, having for its objects the pursuit of natural truth and the permanent welfare of our species, ought to exempt its members from those changeful modes of frivolity which float on the surface of society, and form the atmosphere in which the minor vices delight to play. We believe, indeed, that if a high tone of mind and feeling were general throughout the medical community, a code of etiquette would scarcely be required; and manners and usages peculiar to practitioners of medicine would consist merely in some slight modifications of the ordinary habits of gentlemen, which a few particular positions, fewer perhaps than is usually imagined, render necessary in our own as in all other professions. Again, we are convinced that an improved state of morals and manners would inevitably give rise to a new organization of the profession and a new system of medical politics.

But it is time to notice Mr. Banks. His little book is evidently written in a good spirit, and some of the opinions it contains are judicious; but we would strongly recommend him, in his next edition, to chasten his style and keep closer to his subject; for it must be acknowledged that his remarks are sometimes entirely irrelevant—that certain passages intended to be rhetorical are eminently bombastic and unmeaning—and that others, meant to be humorous, degenerate into mere buffoonery. Thus at p. 16 he expatiates on the baseness of allowing our conscience to be silenced in political matters by considerations of interest. On this there can be but one opinion among honest men; but we cannot perceive that the sentiment applies more to the medical practitioner than to any other person; we therefore deem its expression in this place quite superfluous, while the language in which it is clothed will be found to justify abundantly our charge of inflation. For example:

"The abandoned wretch who prostitutes his political principles to his worldly interests is sunken to the lowest depths of depravity, degradation, and dishonesty; he is a robber and assassin; and would, if he had the power, not only demolish every outpost of all the social institutions of humanity, but with fiendish spirit would strike at the very foundations of that rock on which Justice has built her throne." (p. 16.)

............Pulmo animæ prælargus anhelet!

Again, chap. xx. begins as follows:

"There are a great many trifling questions about which there seems to be no general understanding, and it is to be hoped there never will, as they are quite unworthy of notice. It may perhaps be as well to allude to two or three, just to illustrate our meaning, such as whether prescriptions should be written in English, Latin, Greek, or double Dutch? Whether it be lawful for one medical practitioner to decipher the prescription of another? Whether medical men should dress in black or brown; wear kid or Berlin gloves; eat peas or beans, or go without if they cannot get either?" &c.

All this is very silly, and so far from being worthy of a teacher of medical etiquette, would be far beneath the dignity even of a popular master of the ceremonies.

Mr. Banks's treatise consists of twenty-one chapters, of which the first four are occupied with the relations of medical practitioners to each other, the terms on which they should meet in consultation, and the respective remuneration of the attendant in ordinary and the practitioner called in for the occasion. We shall not enlarge much on these points, because we do not think it possible that they can be satisfactorily adjusted while the organization of the profession remains in its present state. We cannot at all agree with our author that *years* should be made the grand basis on which to found distinctions with respect to rate of remuneration. We do not think that the public would be easily persuaded to set a higher value on the opinion of a stupid man of sixty than on that of a clever man of thirty. At the same time we fully coincide in the popular predilection for the elder man, supposing a parity of abilities, education, and opportunities.

It appears to us that we cannot do better than imitate the French in equalizing the rank of the profession by demanding of all its members the same course of study and the same criteria of proficiency, and disjoining altogether the profession of medicine from the trade or art of the *pharmacien*. In such a state of things, the position of the consulting practitioner (we use the common phrase, *consulted* would be more correct,) ceases to be a point of difficulty. Whoever is called into consultation naturally takes the precedence *on that occasion*, nor do we see any reason why he should not receive a proportionally higher fee. If A takes precedence of B to-day, B may take precedence of A to-morrow. If an individual be very frequently called into consultation, it shows that the profession or the public, perhaps both, have formed a high estimate of his abilities; and he may become gradually a consulting practitioner *par excellence* on a ground of distinction, which ought not to wound the self-love of his brethren; because although it be quite possible that his reputation may be greater in the eyes of the public than of the profession, and may be owing rather to the attraction of his manners or the dexterity of his policy than to any real superiority of professional knowledge or talent, this must always be the case, to a considerable extent, under every possible organization of our own or any other profession—for it arises from the incapacity of men to judge of what they do not understand, and their natural disposition to found confidence on personal predilection where data are wanting for a correct estimate of actual merit.

In chap. vi., "On Mode of Payment," our author insists on the expediency of remunerating all medical practitioners by *fees paid at the time*. To this we entirely assent, in regard to the upper and middle classes of society; nor, as far as concerns them, can we perceive the smallest validity in the objection frequently urged against it. We are told that the bulk of our population could not afford to pay their medical attendant at each visit. The answer is obvious: England is the richest country in the world; there can, therefore, be no reason why the inhabitants of England should not do, in this respect, what is done by those of France, Spain, Italy, and we believe all other European countries. It may also be suggested that if it be not convenient to the patient to fee the doctor at the time, it is probably still less convenient to the latter to wait for his money—for we are a proverbially poor profession all the world over.

In chap. viii., "On Attendance on Professional Persons," Mr. Banks objects to the notion entertained by many that medical practitioners are bound in duty to prescribe gratuitously for their brethren and their families. He admits that there are many circumstances which should call forth our liberality, and induce us to decline the proffered remuneration; but thinks that where one medical man attends another, or the near relative of another, during an illness, etiquette requires that the offer of payment should be made, leaving its acceptance discretionary. We cannot agree with this. One medical man has assuredly no claim to the gratuitous services of another, as a matter either of legal or of moral right; but we think every man endowed with any refinement of feeling would decline remuneration from a professional brother or a member of his household, under all circumstances. When a non-professional person consults a medical practitioner, he does so in the belief that the latter has knowledge and skill in certain matters of which he is himself ignorant, and that the exercise of these, in his behalf, is essential to his well-being and entitled to compensation at his hands; but when one medical practitioner calls for the aid of another, in his own case, a disparity of knowledge and skill cannot, in courtesy at least, be supposed to exist between the two professors of the same art, and we should, therefore, for our own part, always decline any remuneration from a professional brother, because our acceptance of it would seem to place him in the position of one who knew less of medicine than ourselves. This may appear rather a metaphysical view of the subject; but we believe that, on near inspection it will be found to indicate the real point of delicacy—and, as before observed, the question is one of *delicacy*, not of *right*. Again, when we attend a member of the family or household of another practitioner, we attend *in his stead*, and as he would not receive remuneration in such a case, it would be in some sort illogical as well as indelicate to put ourselves on a different footing. With regard to the families of deceased practitioners this reasoning is no longer applicable; but still we think it is a graceful tribute of respect to the memory of one with whom we have been united in the freemasonry of science, to exercise gratuitously towards his nearest kindred the art which he once practised, or should in charity be presumed to have practised, with honour and usefulness. It appears to us, also, that there would be a degree of coarseness, a want of sentiment if not of feeling, in reminding the survivors of their loss, by making our own relation to them less cordial than it would formerly have been. If it be indelicate to accept, it can be scarcely judicious to offer remuneration under the foregoing circumstances. We have indeed known individuals, with more chivalry than judgment, who have been highly offended at such an offer from a fellow-practitioner. This, however, is ridiculous: money is such a precious commodity that when a man offers it to us (unless as a bribe) he must needs have good intentions towards us; and it would therefore be both foolish and ill-natured to be angry with him for so doing.

Chap. ix. treats of "Remuneration for Prescriptions and Medical Certificates." Our author here animadverts justly on the impropriety of a patient's asking a general practitioner to give him the prescription for the medicine he is taking without offering to fee him for it.

This is one of the many mean advantages which vulgar-minded persons are wont to take of the profession, and which the latter is generally too little inclined to resist. Liberality can never consist in allowing ourselves to be cajoled; and we fear that what frequently passes under this name among certain of our brethren, is nothing more than a sneaking submissiveness and dejection of spirit, arising from the conjoint effects of poverty, excessive competition, the consciousness of an anomalous position in society from the union of a profession with a trade, and other causes, which diminish self-respect, and by a necessary consequence encourage impertinent freedom and unreasonable exaction. Mr. Banks further protests against the injustice of expecting practitioners to give certificates relating to health, gratuitously. Here also we, for the most part, agree with him. A medical certificate is, to all intents and purposes, a professional opinion on a case formally delivered in writing; and as such, is as well entitled to a fee, as an opinion orally delivered at the bed-side of a patient. It should also be remembered, that to record in writing, an opinion on the state of health of an individual, is sometimes no pleasant task ; first, because in case of the smallest error of judgment, it lays us open to public animadversion ; secondly, because we may feel ourselves bound in conscience to render a less favorable account of a person's health than he may himself desire ; and hence we run the risk of giving grievous offence, however unreasonable it may be that we should incur wrath, for stating what we believe to be true, when publicly called on to do so. It may be added, that it is not usual for members of other professions to give gratuitous certificates relating to matters with which they are conversant. For all these reasons, we think medical practitioners should not be expected to give certificates without being feed for their trouble, except in the case of poor persons to whom the certificate may be of consequence but who are not in a condition to pay for it.

In chapters xii. and xiii., Mr. Banks considers certain points of professional morality arising out of cases where a practitioner is accidentally sent for to the patient of another, or where a former practitioner has been unhandsomely dismissed. We do not think that any rule can here be of much use, except the great christian law, of doing as one would be done by : whoever adheres to this, and has a moderate degree of judgment to guide his principle, cannot err materially. Our author concludes his remarks on these subjects with a most singular, and if correct, most alarming announcement ; namely, that it is all up with the public morals throughout the world, unless the medical profession take them under their protection ! It appears that governments, hierarchies, and schoolmasters do infinitely more harm than good, and make us worse than we should be without them. The doctor is your only moralist.

"Alas ! thou poor Morality ! If thou art deserted and forsaken by the only men capable of throwing a shield of protection around thee, and infusing new vigour into thy spirit, the members of the medical profession, what hope hast thou ? Nay : despair, and die !" (p. 53.)

We confess we tremble at the approaching alternative of medical supremacy or universal turpitude, and earnestly suggest to our brethren that we should fit ourselves for our high destiny by mending our manners as fast as we can ; for which, to speak honestly, we think there is considerable room.

Chapter xvi. is on "Keeping Shops." Our author here maintains that the circumstance of keeping a shop does not derogate, in any degree, from the personal respectability of a practitioner of medicine. We admit this to be perfectly true with regard to the individual ; at the same time we do not wish to disguise our conviction that shop-keeping is highly derogatory to the profession taken collectively. With the individual, it is often a matter of necessity ; for a young general practitioner, who has nothing but his profession to look to for his maintenance, has scarcely any other alternative; and he may possess, while standing behind his counter, greater talents and acquirements, a more honorable mind, and more elegant manners, than his fortunate neighbour who rejoices in an immunity from many-coloured bottles. But it cannot, we think, be disputed, that an active participation in trade of any kind tends to subvert that frame of mind which befits a member of a learned profession. We apprehend also that it will be long ere the public mind is dispossessed of the prejudice (for such it unquestionably is) which assigns to the shopkeeper a lower station in society than to many another man whose calling may be less useful, and whose personal claims to respect may be far inferior.

Chap. xvii. is on the "Connexion between Practitioners and Chemists." Mr. Banks sees no more impropriety in a physician or other medical practitioner prescribing at a druggist's shop for an annual consideration than in his attending at a dispensary for a fixed salary; and conceives the only difference to be that in the one case he is engaged in the business of a private and in the other of a public dispensary. Neither can Mr. Banks see anything unprofessional in a physician's holding a share in a druggist's business, provided he does so openly and without disguise. He thinks there is no virtual difference between this and his holding a share in a railroad, a bank, or a trading company. There is a show of reason in this; but we think that Mr. Banks has entirely overlooked the real objection to every kind of confraternity between medical practitioners and druggists. This objection we conceive to be that such unions tend to strengthen, in the public mind, that identification of the profession of medicine with the drug-trade which has long exerted so baneful an influence on medicine in this country. It tends in short *to perpetuate the apothecary*, which is the very thing that enlightened men in all classes of the profession wish to abolish. And it tends to perpetuate the evil in a more absurd form than ever: the apothecary, as he now stands, is half doctor and half druggist, which is anomalous enough, yet he is but one man; whereas an association of a physician with a druggist forms a sort of Siamese-twin apothecary—a piece of professional teratology which nobody knows what to make of.

We have already devoted so much space to Mr. Banks's production that we must refrain from commenting on some other subjects on which he has descanted, as that of dispensaries; the conduct to be observed by and towards pupils, assistants, and junior practitioners; solicitation, meaning thereby the beggarly and disreputable practice of leaving cards and calling at houses in the hope of getting patients, &c. These topics are handled by our author with more or less judgment, and we fear we must add with the same occasional discursiveness and intermixture of fustian, which we have had to complain of in those parts of the book which have been more particularly noticed. On the whole, though we cannot compliment Mr. Banks on his talents as an author, we may nevertheless recommend his work to the perusal of our readers. Its general spirit is praiseworthy, and the adoption of some of its precepts would materially improve the state of the profession.

